# Sonographic Evaluation of Muscle Echogenicity for the Detection of Intensive Care Unit-Acquired Weakness: A Pilot Single-Center Prospective Cohort Study

**DOI:** 10.3390/diagnostics12061378

**Published:** 2022-06-02

**Authors:** Felix Klawitter, Uwe Walter, Robert Patejdl, Josefine Endler, Daniel A. Reuter, Johannes Ehler

**Affiliations:** 1 Department of Anesthesiology and Intensive Care Medicine, University Medical Center Rostock, 18057 Rostock, Germany; josefine.endler@uni-rostock.de (J.E.); daniel.reuter@med.uni-rostock.de (D.A.R.); johannes.ehler@med.uni-rostock.de (J.E.); 2 Department of Neurology, University Medical Center Rostock, 18147 Rostock, Germany; uwe.walter@med.uni-rostock.de; 3 Oscar Langendorff Institute of Physiology, University Medical Center Rostock, 18057 Rostock, Germany; robert.patejdl@uni-rostock.de

**Keywords:** diagnostic ultrasound, ICU-AW, muscle echogenicity, muscle weakness

## Abstract

Qualitative assessment by the Heckmatt scale (HS) and quantitative greyscale analysis of muscle echogenicity were compared for their value in detecting intensive care unit-acquired weakness (ICU-AW). We performed muscle ultrasound (MUS) of eight skeletal muscles on day 3 and day 10 after ICU admission. We calculated the global mean greyscale score (MGS), the global mean z-score (MZS) and the global mean Heckmatt score (MHS). Longitudinal outcome was defined by the modified Rankin scale (mRS) and Barthel index (BI) after 100 days. In total, 652 ultrasound pictures from 38 critically ill patients (18 with and 20 without ICU-AW) and 10 controls were analyzed. Patients with ICU-AW had a higher MHS on day 10 compared to patients without ICU-AW (2.6 (0.4) vs. 2.2 (0.4), *p* = 0.006). The MHS was superior to ROC analysis (cut-off: 2.2, AUC: 0.79, *p* = 0.003, sensitivity 86%, specificity 60%) in detecting ICU-AW compared to MGS and MZS on day 10. The MHS correlated with the Medical Research Council sum score (MRC-SS) (r = −0.45, *p* = 0.004), the mRS (r = 0.45; *p* = 0.007) and BI (r = −0.38, *p* = 0.04) on day 100. Qualitative MUS analysis seems superior to quantitative greyscale analysis of muscle echogenicity for the detection of ICU-AW.

## 1. Introduction

Muscle ultrasound (MUS) is a promising technique for the non-invasive detection of neuromuscular diseases, especially in critically ill patients who are at high risk of developing intensive care unit-acquired weakness (ICU-AW) [1,2,3,4,5]. Among different ultrasound parameters, the ultrasonographic depiction of muscle tissue brightness, called muscle echogenicity (ME), has also been evaluated in ICU patients [6,7,8,9]. Increased ME has been proven to correlate with histological alterations such as myofiber necrosis and fascial inflammation [10]. ME can be assessed quantitatively by calculating the mean greyscale value of a defined muscle area within an ultrasound image [11], or alternatively by using the qualitative Heckmatt scale (HS), which grades the muscle and bone cortical echo signal according to brightness and structural homogeneity [12,13,14]. The HS seems suitable to identify patients with critical illness polyneuromyopathy [15], whereas software-based greyscale analysis is still under extensive evaluation for neuromuscular disorders [16,17,18] and recently in patients with COVID-19 [19,20]. However, both assessment methods have never been compared in ICU patients at risk of ICU-AW. Furthermore, evidence of a correlation with muscle strength and the functional outcome in patients with ICU-AW is still lacking. The aim of this observational study is to compare the diagnostic value of qualitative and quantitative assessments of ME to identify critically ill patients with ICU-AW. We hypothesize that both ultrasound method mean values correlate significantly with clinical scores used in the detection of ICU-AW.

## 2. Materials and Methods

### 2.1. Study Design, Inclusion and Exclusion Criteria

Ultrasound data were derived from a prospective combined experimental and clinical study evaluating new approaches to diagnose ICU-AW in intensive care patients at two perioperative ICUs of a single university hospital. The study was approved by the local ethics committee of the University of Rostock in January 2016 (ethics identifier: AS 2016-0016). The study was registered as a clinical trial (ClinicalTrials.gov: NCT02706314). The results of the experimental study part have already been published [21]. Written informed consent for participation and data collection was given by the patient or a legal representative before study inclusion. Inclusion criteria comprised patients ≥ 18 years (female and male patients) with a Sequential Organ Failure Assessment (SOFA) score ≥ 8 on three consecutive days within the first five days after ICU admission [22]. Exclusion criteria comprised any pre-existing neuromuscular disease, participation in another study and high-dose corticosteroid treatment (≥300 mg hydrocortisone or equivalent per day). Study assessments (clinical examination and MUS) were performed on day 3 and day 10 after study inclusion. Healthy controls were included after written informed consent. To assess functional outcomes, we evaluated the Barthel index (BI) as well as the modified Rankin scale (mRS) 100 days after study inclusion in each patient. Due to the fact that the present study was designed as a pilot study, a power calculation was not performed.

### 2.2. Assessment of Intensive Care Unit-Acquired Weakness

The Medical Research Council sum score (MRC-SS) was assessed on day 3 and day 10 by an ICU staff member (FK) who was blinded to the results of the MUS. Before clinical examination, analgo-sedation was interrupted, if present. Patients were eligible for assessment if five initial commands were carried out sufficiently (1. “Open and close eyes”, 2. “Look at me”, 3. “Open your mouth and put your tongue out”, 4. “Nod your head”, 5. “Raise your eyebrows when I have counted up to five”). According to current recommendations, patients were diagnosed as ICU-AW positive (ICU-AW+) with an MRC-SS < 48 and ICU-AW negative (ICU-AW-) with an MRC-SS ≥ 48 on day 10 [23]. 

### 2.3. Muscle Ultrasound Protocol 

Ultrasound examinations were performed by a consultant neurologist with 15 years of experience in MUS (UW) using a standardized in-house protocol (see Supplementary File S1). The examiner was blinded to the results of the clinical examination. For image acquisition, an Aplio 300 Toshiba ultrasound system (Canon; Tokyo, Japan) equipped with a 4–14 MHz linear array transducer was used. The predefined musculoskeletal setup with constant image gain and contrast was selected. Imaging depth and focus position were adapted during the ultrasound procedure. Three and 10 days after ICU admission, the following muscles were bilaterally assessed by MUS: biceps brachii (BB), brachioradialis (BR), rectus femoris of the quadriceps femoris (QF) and tibialis anterior (TA). 

### 2.4. Qualitative Assessment of Muscle Echogenicity Using the Heckmatt Scale

Echogenicity of each muscle was rated visually by using the four-grade HS as previously described [12]: grade 1 = normal echo-intensity of the muscle with normal echo-intensity and full visualization of the adjacent bone cortex; grade 2 = increased echo-intensity of the muscle with normal echo-intensity and full visualization of the adjacent bone cortex; grade 3 = marked increased echo-intensity of the muscle with altered echo-signal and incomplete visualization of the adjacent bone cortex; grade 4 = strongly increased echo-intensity of the muscle with complete loss of bone cortex echo-signal. The global mean Heckmatt score (MHS) was calculated by averaging all HSs of each single muscle in every individual study participant on days 3 and 10.

### 2.5. Quantitative Greyscale Analysis of Muscle Echogenicity with ImageJ

A trained research assistant (JosE) performed the greyscale analysis using the software ImageJ (Version 1.52a, Wayne Rasband, National Institute of Health, USA) and was blinded to the results of MUS and clinical examination. Prior to greyscale analysis using the histogram function, all images were converted into 8-bit greyscale images. A region of interest (ROI) was defined by marking the maximum visible cross-sectional area (CSA) of the muscle of interest excluding surrounding muscle fascia. Prominent intramuscular fascia and image artefacts were excluded from the ROI. As muscle CSA was shown to correlate with muscle strength [24], we defined the maximum visible muscle CSA to be the most appropriate parameter for a correlation analysis between ME and muscle strength. To compare the echogenicity of different muscles, the z-score was calculated for each mean greyscale value, as described by [25] using the equation:


$$z-score\;\left(SD\right)=\frac{\left(measured\;value\;\left[patients\right]-normal\;value\left[controls\right]\right)}{standard\;deviation\;of\;normal\;value\;\left[controls\right]}$$

The greyscale level standard deviation (GSSD) within the ultrasound image was measured to determine muscle tissue homogeneity [17]. The global mean greyscale score (MGS) and the global mean z-score (MZS) were calculated as described above by averaging the greyscale values and the z-scores of all muscles within the ICU-AW+ and ICU-AW− patients for days 3 and 10. The subcutaneous fat layer thickness (SFT) was recorded by measuring the distance from the cutis to the upper muscle fascia perpendicular to the cortical bone. 

### 2.6. Statistical Analysis

For statistical analysis, we used MS-Excel 2010 (Microsoft, Redmond, WA, USA) and IBM SPSS Statistics (Version 25, IBM Corp., Armonk, NY, USA). Data are presented as sum (percent) or mean (standard deviation). Student’s *t*-test was used for normally distributed data, and the Mann–Whitney *U*-test to compare continuous or discrete variables. The Chi square test with Yates correction was used for categorical variables. In case of expected values of <5 in the 2 × 2 contingency table, Fisher‘s exact test was performed. The Pearson correlation coefficient was calculated to compare normally distributed variables; otherwise, we used the Spearman rank correlation coefficient. Receiver operating characteristics (ROC) with a 95% confidence interval were calculated to determine the discriminative ability of ME to detect ICU-AW. Statistical significance was indicated by *p* < 0.05. All statistical tests were two-sided. Since eight muscles were compared, a Bonferroni correction was employed with the significance level set at *p* ≤ 0.006 for the referring group comparisons.

## 3. Results

### 3.1. Study Population Characteristics

**Figure 1:**
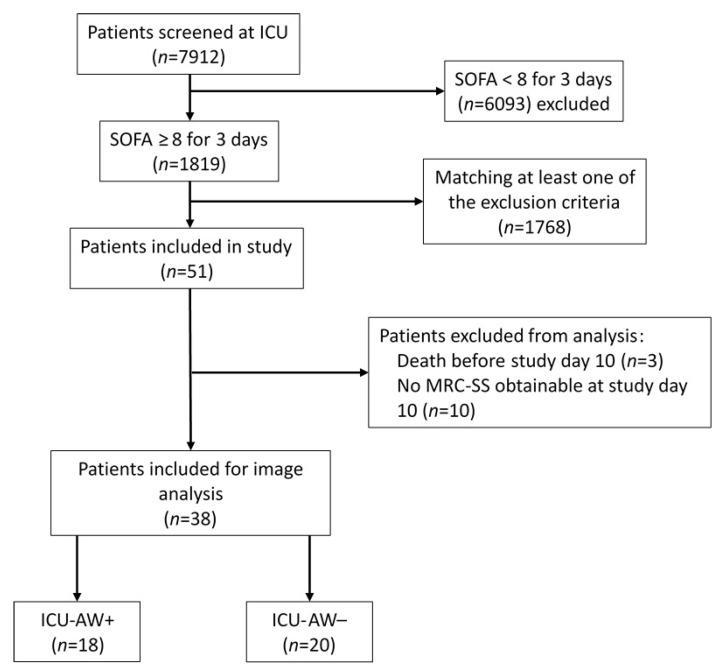
Flowchart of patient screening and study cohort inclusion. Feasibility of MRC-SS testing was checked by using standardized requests the patient had to follow: “Open and close your eyes”, “Look at me”, “Open your mouth and put out your tongue“, “Nod your head”, “Raise your eyebrows until I have counted to five”. ICU-AW was assumed with an MRC-SS < 48. ICU-AW+: patients with intensive care unit-acquired weakness. ICU-AW−: patients without intensive care unit-acquired weakness. ICU: intensive care unit. MRC-SS: Medical Research Council sum score. SOFA: Sequential Organ Failure Assessment score.

We enrolled 51 postoperative critically ill patients and 10 healthy controls (Figure 1). Three patients died before day 10 and were therefore excluded. The MRC-SS could not be obtained in 10 patients on day 10 due to persistent impairment of consciousness. In a detailed neurological examination, most patients (9/10) presented typical clinical findings of a generalized neuromuscular dysfunction (symmetric flaccid palsy, hypo- to areflexia and reduced or absent muscle tone in all tested muscles), congruent with the symptoms of an ICU-AW. However, because of the impossibility of obtaining an MRC-SS value, these ten patients were also excluded from further analysis. Finally, 38 critically ill patients were included for image analysis (18 patients with ICU-AW and 20 patients without ICU-AW). Table 1 shows the baseline characteristics of the study population.

### 3.2. Quantitative Greyscale Analysis of Muscle Echogenicity in Patients with and without ICU-AW Compared to Controls

In total, 652 ultrasound images of 38 critically ill patients and 10 healthy controls were analyzed. Mean values and statistical data are summarized in Table 2 (corresponding *p* values available in Supplementary File S2). The calculated MGS of ICU-AW+ (day 3: 75.5 (21.8); day 10: 72.1 (24.2)) and ICU-AW− (day 3: 73.2 (30.0); day 10: 69.2 (27.5)) patients was higher compared to the controls, with 49.5 (12.8) on days 3 (ICU-AW+ *p* = 0.002; ICU-AW− *p* = 0.02) and 10 (ICU-AW+ *p* = 0.01; ICU-AW− *p* = 0.04).

### 3.3. Qualitative Ultrasound Assessment of Muscle Echogenicity in Patients with and without ICU-AW Compared to Controls 

Patients with and without ICU-AW had higher HS gradings compared to the control group on days 3 and day 10 (Table 2). Furthermore, the calculated MHS of ICU-AW+ (day 3: 2.6 (0.5); day 10: 2.6 (0.4)) and ICU-AW− (day 3: 2.4 (0.4); day 10: 2.2 (0.4)) patients was higher compared to controls at on days 3 (1.1 (0.1), *p* < 0.001) and 10 (1.1 (0.1), *p* < 0.001).

### 3.4. Quantitative Greyscale Analysis of Muscle Echogenicity Inpatients with and without ICU-AW

**Figure 2:**
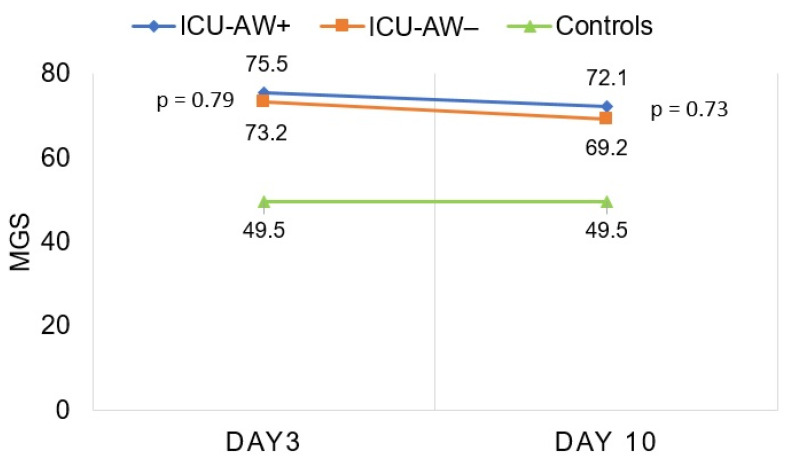
Longitudinal assessment of the MGS in ICU-AW+ patients, ICU-AW− patients and healthy controls on days 3 and 10. ICU-AW: intensive care unit-acquired weakness. ICU-AW+: patients with intensive care unit-acquired weakness. ICU-AW−: patients without intensive care unit-acquired weakness. ICU: intensive care unit. MGS: global mean greyscale score.

We found no differences in the greyscale values of any extremity muscle on day 3 or day 10 between ICU-AW+ and ICU-AW− patients (Table 2, corresponding *p* values of group comparisons are available in Supplementary File S2). Furthermore, there were no differences in the z-score, the GSSD or the SFT of any examined muscle between both groups on days 3 and 10. There were also no differences in the MGS (Figure 2, day 3: 75.5 (21.8) vs. 73.2 (30.0), *p* = 0.79; day 10: 72.1 (24.2) vs. 69.2 (27.5), *p* = 0.73), or in the MZS (day 3: 1.0 (0.8) vs. 0.9 (1.1), *p* = 0.73; day 10: 0.8 (0.9) vs. 0.7 (1.0), *p* = 0.73) between patients with and without ICU-AW.

### 3.5. Qualitative Ultrasound Assessment of Muscle Echogenicity in Patients with and without ICU-AW

**Figure 3:**
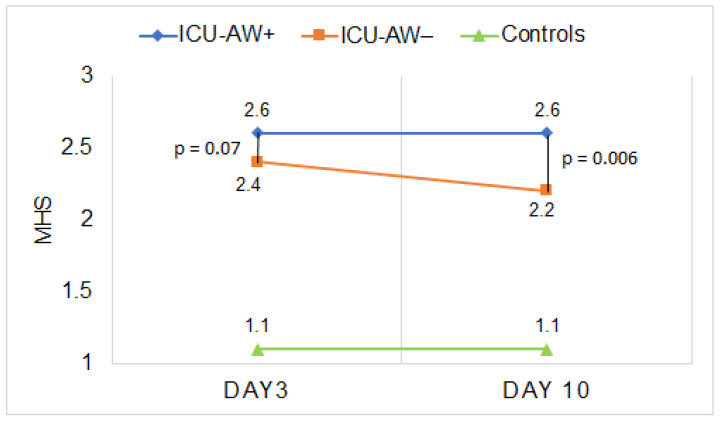
Longitudinal assessment of the MHS in ICU-AW+ patients, ICU-AW− patients and healthy controls on days 3 and 10. ICU-AW: intensive care unit-acquired weakness. ICU-AW+: patients with intensive care unit-acquired weakness. ICU-AW−: patients without intensive care unit-acquired weakness. ICU: intensive care unit. MHS: global mean Heckmatt score.

We found no differences in most of the HS grading of single muscles between ICU-AW+ and ICU-AW− patients on day 3 (Table 2). There was also no difference in the MHS (Figure 3, Table 3, 2.6 (0.4) vs. 2.4 (0.4), *p* = 0.07) on day 3 (all corresponding *p* values are available in Supplementary File S2). In contrast, the MHS on day 10 was significantly higher in ICU-AW+ patients compared to ICU-AW− patients (Table 3: 2.6 (0.4) vs. 2.2 (0.5), *p* = 0.006).

### 3.6. Diagnostic Value of Muscle Echogenicity and Relationship to Functional Patient Outcome

**Figure 4:**
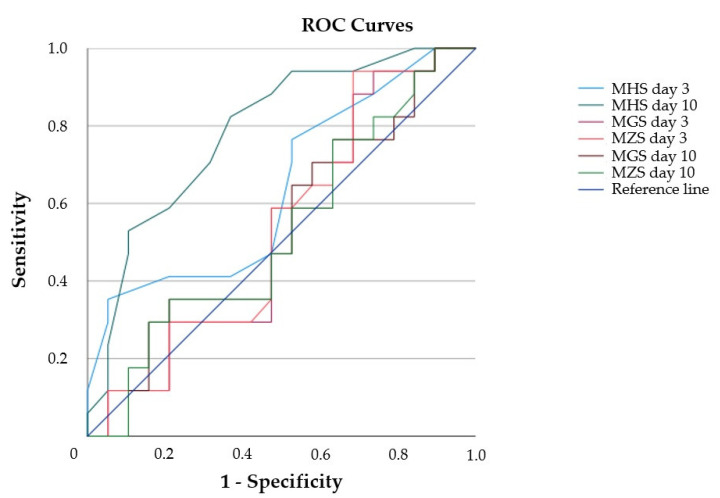
ROC curves of ultrasonographic parameters on study days 3 and 10. MGS: global mean greyscale score. MHS: global mean Heckmatt score. MZS: global mean z-score. ROC: receiver operating characteristics.

To determine the diagnostic value of the parameter ME in MUS for the differentiation of ICU-AW+ and ICU-AW− patients, we performed a ROC analysis of the MGS, MZS and MHS for both assessments on days 3 and 10 (Figure 4, Table S4 in the Supplementary File S2). Only the MHS on day 10 differentiated between ICU-AW+ and ICU-AW− patients, and the ROC analysis proved statistical significance (*p* = 0.003). Furthermore, using the MHS, we calculated an optimal cut-off value of 2.2 on day 10 with a sensitivity of 86% and a specificity of 60% to identify ICU-AW+ patients.

**Figure 5:**
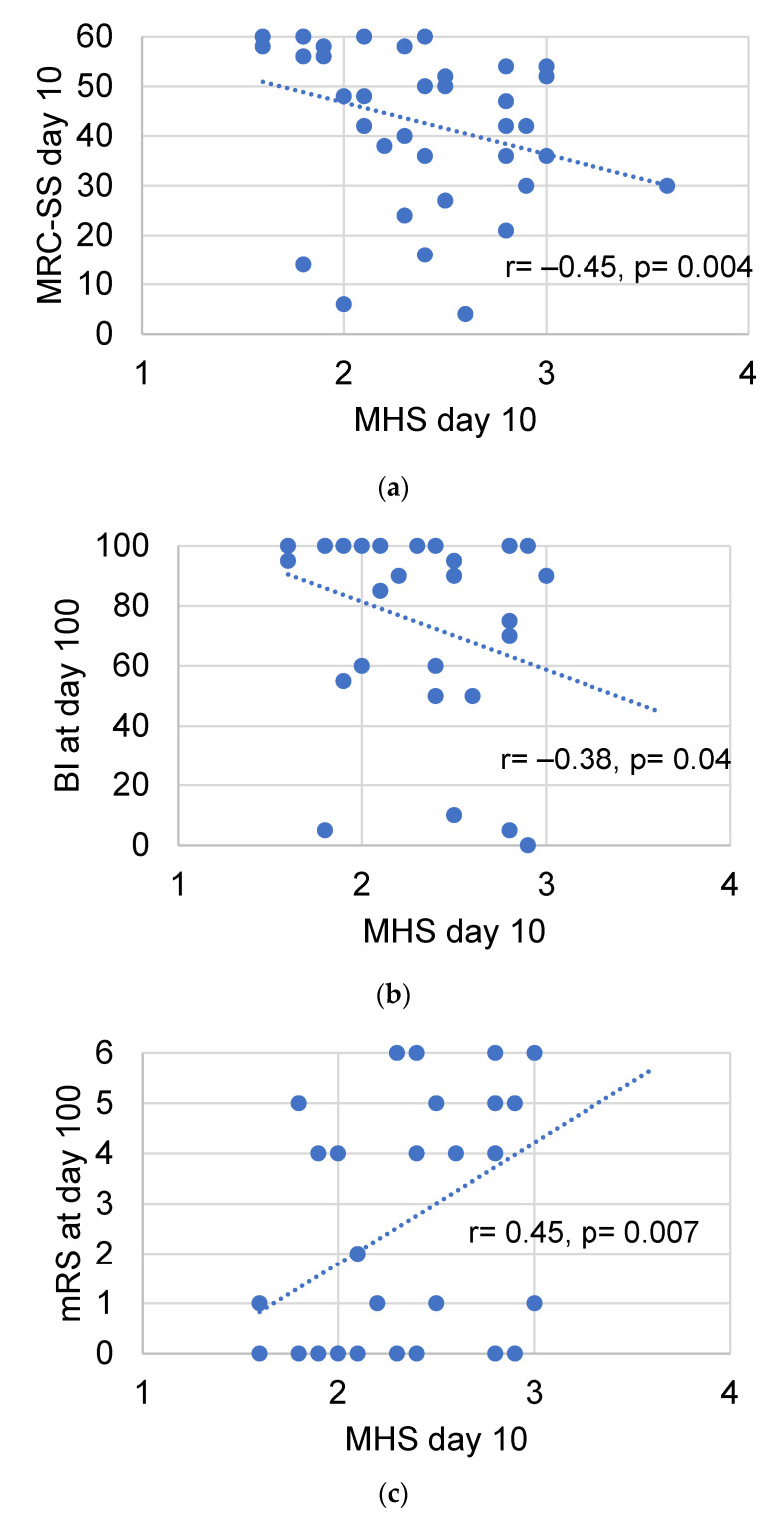
Correlation of the MHS with clinical outcome parameters. Correlation of the MHS on day 10 with the MRC-SS on day 10 (**a**), the BI (**b**) and the mRS on day 100 (**c**). BI: Barthel index. MHS: global mean Heckmatt score. MRC-SS: Medical Research Council sum score. mRS: Modified Rankin scale. r: correlation coefficient.

We found no correlation between the MRC-SS on day 3 and the corresponding MGS (*p* = 0.85), MZS (*p* = 0.34) or MHS (*p* = 0.05) in the population of ICU patients. On day 10, the MHS correlated with the corresponding MRC-SS (Figure 5a: r = −0.45, *p* = 0.004), but not the MGS (*p* = 0.90) or the MZS (*p* = 0.89). An increased MHS on day 10 was related to a decrease of the BI (Figure 5b: r = −0.38, *p* = 0.04) and an increase of the mRS (Figure 5c: r = 0.45, *p* = 0.007) on day 100 after study inclusion. In contrast, neither the MGS (BI: *p* = 0.25; mRS: *p* = 0.97) nor the MZS (BI: *p* = 0.26; mRS: *p* = 0.97) on day 10 correlated with functional outcome parameters.

## 4. Discussion

Our study compared the utility of two different ultrasound methods to assess ME in critically ill patients at risk of ICU-AW. Specifically, we compared software-based greyscale image analysis and visual qualitative HS in this population. Our results indicate that both methods are suitable for the detection of increased ME in skeletal muscles of critically ill patients compared to healthy controls. However, in this study, only the summarized qualitative assessment of the echo intensity in multiple skeletal muscles represented by the MHS was able to identify patients with ICU-AW with good accuracy, and correlated with the severity of muscular weakness and functional impairment 100 days after study inclusion.

**Figure 6:**
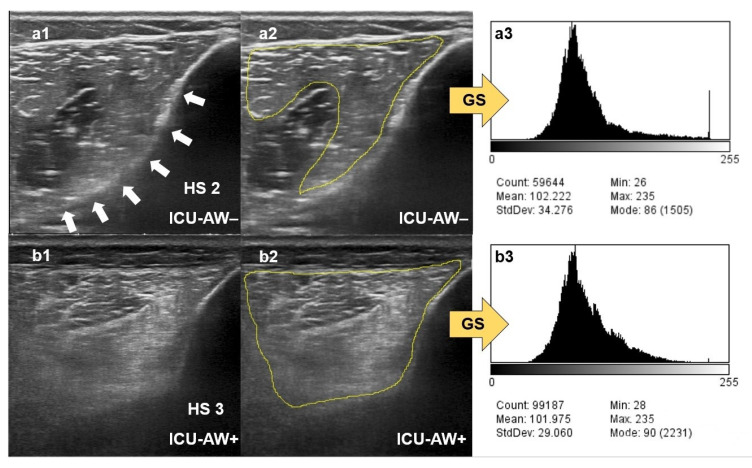
Differences in assessment of muscle echogenicity. (**a1**) Qualitative assessment of muscle echogenicity of the tibialis anterior muscle of an ICU-AW− patient with increased muscle echo intensity, but nearly fully visible cortical bone echo signal (white arrows), rated with the Heckmatt scale (HS). (**a2**) Quantitative assessment of muscle echogenicity of the same muscle depicted in a1 using ImageJ by selecting a region of interest (yellow marked; the acoustic shadow image artifact behind the central tibialis anterior aponeurosis has been excluded) within the affected muscle and consecutive greyscale analysis (GS) with the corresponding histogram (**a3**). (**b1**) Qualitative assessment of muscle echogenicity of the tibialis anterior muscle of an ICU-AW+ patient with increased muscle echo intensity and nearly vanished cortical bone echo signal, rated HS 3. (**b2**) Quantitative assessment of muscle echogenicity of the same muscle depicted in b1 using ImageJ by selecting a region of interest (yellow marked) within the affected muscle and consecutive GS with the corresponding histogram in (**b3**). The mean GS values of the quantitative assessments (**a3**,**b3**) are almost equal, so patients with ICU-AW cannot be distinguished from patients without ICU-AW. In contrast, in the qualitative assessments (**a1**,**b1**) using the HS, a clear difference in the visibility of the tibialis cortical bone echo signal between ICU-AW+ and ICU-AW- patients can be seen.

Increased ME has been consistently reported in studies employing MUS in critically ill patients with neuromuscular dysfunction compared to ICU patients without polyneuromyopathy or healthy controls [9,10,15,26,27]. Our study confirms these earlier findings. In addition, it indicates that the assessment of increased ME graded by the HS in multiple skeletal muscles at the same time can discriminate critically ill patients with ICU-AW from those without ICU-AW. An MHS of 2.2 on day 10 predicts ICU-AW with a sensitivity of 86% and a specificity of 60%. Similar results were reported by Kelmenson et al., who calculated a sensitivity of 82% and a specificity of 57% for a HS > 2 in any muscle to predict critical illness polyneuromyopathy [15]. Here, ultrasonographic assessment of ME in multiple different skeletal muscles within a combined approach seems superior to the evaluation of single muscles only, since the generalized nature of a systemic neuromuscular dysfunction can be depicted more precisely. Surprisingly, we found no differences of muscle greyscale values or corresponding z-scores between ICU-AW+ and ICU-AW− patients. As visualized in Figure 6, an explanation for our results might be found in the method itself. Quantitative greyscale analysis simply assesses the “brightness” of pixels within muscle tissue, while the HS incorporates the grading of both muscle and cortical bone echo texture [11,12]. Increased ME in critically ill patients with acute neuromuscular dysfunction occurs because of changes in muscle architecture and fiber composition due to the development of intramuscular necrosis and inflammation, rather than due to volume overload and tissue edema [10]. Therefore, muscle tissue with higher impedance values reflects more ultrasound waves, which cannot penetrate into deeper tissues compared to muscle tissue from healthy controls. This finally results in a darker sonographic depiction of tissues underlying the affected muscle, e.g., the cortical bone. Therefore, the evaluation of echogenicity and echo texture of surrounding tissues seems to provide important additional information in the rating of muscular ultrasound images [12].

A decline in muscle GSSD was associated with an increase of muscle homogeneity due to inflammation and muscle fiber breakdown [26]. However, we could not observe any differences within the GSSD between the groups of ICU patients, indicating similar muscle tissue homogeneity. Alterations in muscle architecture occur early within the first hours of critical illness and then persist in an ongoing process of muscle protein breakdown and muscle fiber derangement [7]. We speculate that muscle damage had already reached a high level before the first ultrasound examination, so no further changes were detectable. In contrast, we observed a positive correlation between changes in ME assessed by the HS and previous inflammation serum biomarkers [28].

Although a possible association of increased ME values with a decline in muscle strength and function contributing to the development of an acquired muscle dysfunction has been hypothesized [9,26], this could not be clearly confirmed. Former studies showed a negative correlation between a gain in ME and isometric muscle force in healthy elderly people [29,30,31]. For the critically ill, Parry et al. also reported increased greyscale levels in patients with impaired muscle function and strength, but did not compare patients with and without ICU-AW [32]. We observed a significant negative correlation between increased ME and decreased muscle strength assessed by the MRC-SS on day 10 in all ICU patients for the qualitative assessment using the MHS, but not for the MGS.

Pillen and co-workers compared the quantitative and qualitative assessment of ME in 76 children with neuromuscular diseases [25]. Sensitivity and interobserver reliability were higher with the quantitative approach and the authors concluded that greyscale analysis is superior to the HS in ME grading. However, these findings were never confirmed for adult perioperative ICU patients with newly acquired neuromuscular dysfunction. 

Muscle echogenicity was reported to correlate with age [32,33] and female sex [34,35]. In the present study, age was not significantly different between ICU-AW+ and ICU-AW− patients, but it was different between the cohort of critically ill patients and the healthy controls. We observed no difference in the distribution of sex between controls and ICU patients. Grimm et al. reported higher ME values in intensive care patients compared to age-matched healthy controls, not indicating a relevant interference between age and ME in this study population [9]. Furthermore, due to a comparable distribution of female participants within both groups of our study population, a gender-based bias is unlikely. A positive correlation between the SFT and increased ME in MUS was reported as a potential confounder in various non-ICU studies [30,35,36]. In contrast, Cartwright investigated MUS in 16 ICU patients and found an increase in ME only in muscles with a constant SFT within the whole observational period [26]. Consistent with this, our results do not support an interference of the SFT with ME in MUS, since no differences for the SFT of corresponding muscles could be obtained between the ICU-AW+ and ICU-AW− patients. 

Some limitations of the present study must be mentioned. The study was designed as a pilot study without initial power calculation. Due to our strict exclusion criteria, only 51 patients were consecutively enrolled in this single-center study and data from just 38 patients were available to image analysis. In 10 patients, the MRC-SS could not be assessed on day 10. This represents a typical shortcoming in the daily clinical routine of simply using the MRC-SS to detect ICU-AW in critically ill patients, which underlines the diagnostic advantage of MUS in assessing patients independently from their ability to cooperate. The controls were not matched for age, so they were significantly younger than the ICU patients, possibly affecting ME. Despite careful conduct of the ultrasound examinations, changes in ME by unintentional slight tilting of the ultrasound probe cannot be fully ruled out. The visual assessment of ME is assumed to be more subjective, resulting in a moderate inter- and intra-observer agreement [25]. Therefore, a highly experienced examiner was selected to perform the ultrasound examinations in the present study, whereas the applicability for non-expert clinicians in daily clinical routine remains to be evaluated in further studies. 

## 5. Conclusions

Our findings support the use of a comprehensive muscular ultrasound protocol for the assessment of increased ME in critically ill patients to detect ICU-AW. In the present study, software-based greyscale analysis was not able to distinguish between patients with and without ICU-AW. We provide evidence that qualitative ultrasound imaging-based scores comprising several diagnostic criteria may be superior in detecting ME compared with a pure qualitative method. Future studies are warranted to verify the prognostic value of this valuable diagnostic tool if applied by less experienced, adequately trained investigators.

## Figures and Tables

**Table 1:** Study population characteristics. Statistically significant *p* values (*p* < 0.05) are printed in bold. The results are rounded to the first decimal place. APACHE II: Acute Physiology and Chronic Health Evaluation Score II. BMI: body mass index. ICU-AW: intensive care unit-acquired weakness. ICU: intensive care unit. MRC-SS: Medical Research Council sum score. mRS: modified Rankin scale. N/A: value not available. SD: standard deviation. SOFA: Sequential Organ Failure Assessment score.

	Healthy Controls	ICU-AW−	ICU-AW+	*p* Value (ICU-AW − vs. Healthy Controls)	*p* Value (ICU-AW+ vs. Healthy Controls)	*p* Value (ICU-AW+ vs. ICU-AW−)
Total (%)	10 (26.3)	20 (41.7)	18 (37.5)	N/A	N/A	N/A
Female (%)	4 (40)	5 (25)	8 (44.4)	0.43	1	0.11
Age, years (SD)	53.1 (8.0)	68.1 (14.2)	70.8 (11.5)	**0.002**	**<0.0001**	0.52
BMI, kg/cm^2^ (SD)	27.3 (4.9)	28.4 (5.1)	28.6 (5.9)	0.56	0.56	0.94
APACHE II (SD)	N/A	23.7 (6.2)	26.2 (3.7)	N/A	N/A	0.14
SOFA day 3 (SD)	N/A	10.1 (2.3)	12.9 (2.8)	N/A	N/A	**0.001**
SOFA day 10 (SD)	N/A	3.6 (3.0)	6.6 (2.8)	N/A	N/A	**0.001**
MRC-SS day 3 (SD)	60 (0)	51.2 (12.4)	30.5 (8.7)	**<0.0001**	**<0.0001**	**0.01**
MRC-SS day 10 (SD)	60 (0)	55.4 (4.2)	29.5 (12.9)	**<0.0001**	**<0.0001**	**<0.0001**
mRS 100 days after study inclusion (SD)	0 (0)	2.1 (2.6)	3.1 (2.2)	**0.005**	**<0.0001**	0.32
Barthel index before ICU admission (SD)	N/A	97.5 (4.4)	93.9 (16.4)	N/A	N/A	0.68
Barthel index 100 days after study inclusion (SD)	N/A	87.9 (25.5)	63.7 (36.0)	N/A	N/A	**0.03**
28-day survival (%)	10 (100)	18 (90)	18 (100)	0.54	1	0.49
100-day survival (%)	10 (100)	16 (80)	16 (88.9)	0.27	0.52	0.66

**Table 2:** Parameters of muscle echogenicity. Data presented as mean (standard deviation). Corresponding *p* values of group comparisons are available in Supplementary File S2. The results are rounded to the first decimal place. GS: greyscale value. GSSD: greyscale standard deviation. HS: Heckmatt scale. ICU-AW: intensive care unit-acquired weakness. N/A: value not available. SFT: subcutaneous fat layer thickness.

	Healthy Controls	ICU-AW+ Day 3	ICU-AW− Day 3	ICU-AW+ Day 10	ICU-AW− Day 10
**Biceps brachii left**					
GS	49.4 (19.7)	71.8 (25.3)	66.4 (24.3)	73.9 (21.5)	63.4 (26.8)
z-Score	N/A	0.9 (1.0)	0.7 (0.9)	0.9 (0.8)	0.5 (1.0)
HS	1.0 (0.0)	2.6 (0.6)	2.2 (0.5)	2.4 (0.6)	2.0 (0.7)
GSSD	26.0 (4.4)	27.4	25.9	27.8	28.5
SFT, cm	0.5 (0.1)	0.6 (0.2)	0.6 (0.2)	0.7 (0.3)	0.6 (0.2)
**Biceps brachii right**					
GS	44.8 (17.2)	74.7 (31.2)	76.5 (38.0)	70.5 (31.4)	65.8 (32.2)
z-Score	N/A	1.1 (1.1)	1.1 (1.4)	0.9 (1.1)	0.8 (1.2)
HS	1.0 (0.0)	2.5 (0.5)	2.3 (0.7)	2.3 (0.8)	2.0 (0.7)
GSSD	27.9 (3.5)	24.6	24.3	25.3	27.1
SFT, cm	0.5 (0.1)	0.7 (0.2)	0.6 (0.1)	0.7 (0.3)	0.6 (0.2)
**Brachioradialis left**					
GS	41.6 (17.4)	71.7 (25.6)	74.5 (35.8)	64.9 (24.1)	62.5 (30.7)
z-Score	N/A	1.2 (1.0)	1.3 (1.4)	0.9 (0.9)	0.8 (1.2)
HS	1.0 (0.0)	2.5 (0.6)	2.3 (0.4)	2.4 (0.5)	2.0 (0.7)
GSSD	25.6 (5.0)	27.8	25.1	26.5	26
SFT, cm	0.5 (0.1)	0.6 (0.2)	0.6 (0.1)	0.6 (0.2)	0.5 (0.1)
**Brachioradialis right**					
GS	44.4 (10.2)	74.6 (32.3)	67.4 (35.2)	67.4 (31.0)	62.3 (26.5)
z-Score	N/A	1.2 (1.3)	0.9 (1.4)	0.9 (1.3)	0.7 (1.1)
HS	1.0 (0.0)	2.6 (0.6)	2.3 (0.6)	2.4 (0.7)	1.9 (0.6)
GSSD	24.4 (3.9)	27.3	25.3	25.8	27.7
SFT, cm	0.5 (0.1)	0.6 (0.2)	0.6 (0.2)	0.6 (0.2)	0.5 (0.2)
**Rectus femoris of the quadriceps femoris left**					
GS	56.7 (26.2)	71.4 (16.7)	77.6 (31.4)	73.5 (20.0)	72.4 (30.8)
z-Score	N/A	0.5 (0.6)	0.7 (1.1)	0.6 (0.7)	0.6 (1.1)
HS	1.3 (0.5)	2.8 (0.7)	2.4 (0.5)	2.6 (0.6)	2.3 (0.8)
GSSD	28.1 (8.4)	25.1	25.1	26.3	27.6
SFT, cm	0.7 (0.2)	1.1 (0.5)	0.9 (0.4)	1.1 (0.5)	0.9 (0.3)
**Rectus femoris of the quadriceps femoris right**					
GS	63.7 (7.7)	69.8 (21.3)	71.7 (30.9)	73.2 (25.5)	73.4 (27.6)
z-Score	N/A	0.2 (0.7)	0.3 (1.0)	0.3 (0.8)	0.3 (0.9)
HS	1.1 (0.3)	2.6 (0.7)	2.4 (0.6)	2.6 (0.5)	2.4 (0.6)
GSSD	31.2 (3.8)	25.4	23.6	28.4	27.4
SFT, cm	0.7 (0.2)	1.1 (0.6)	0.9 (0.4)	1.1 (0.5)	0.9 (0.4)
**Tibialis anterior left**					
GS	49.1 (14.3)	74.4 (25.0)	83.7 (30.5)	80.6 (36.1)	75.9 (34.4)
z-Score	N/A	1.0 (1.0)	1.3 (1.2)	1.2 (1.4)	1.0 (1.3)
HS	1.1 (0.3)	2.9 (0.7)	2.6 (0.6)	2.7 (0.5)	2.5 (0.5)
GSSD	25.1 (6.1)	26.6	25.1	26.1	29.7
SFT, cm	0.4 (0.2)	0.5 (0.3)	0.4 (0.2)	0.5 (0.3)	0.5 (0.3)
**Tibialis anterior right**					
GS	46.8 (13.9)	85.9 (27.7)	76.4 (29.8)	77.7 (31.8)	79.9 (30.1)
z-Score	N/A	1.4 (1.0)	1.1 (1.1)	1.1 (1.2)	1.2 (1.1)
HS	1.2 (0.4)	2.7 (0.8)	2.7 (0.8)	2.9 (0.5)	2.3 (0.6)
GSSD	27.4 (4.0)	29	27.6	27	28.1
SFT, cm	0.4 (0.2)	0.5 (0.3)	0.4 (0.2)	0.6 (0.3)	0.4 (0.2)

**Table 3:** Global muscle echogenicity parameters. Data presented as mean (standard deviation). The results are rounded to the first decimal place. ICU-AW: intensive care unit-acquired weakness. MGS: global mean greyscale score. MHS: global mean Heckmatt score. MZS: global mean z-score. N/A: data not available.

	Healthy Controls	ICU-AW+ Day 3	ICU-AW− Day 3	ICU-AW+ Day 10	ICU-AW− Day 10
MGS	49.5 (12.8)	75.5 (21.8)	73.2 (30.0)	72.1 (24.2)	69.2 (27.5)
MZS	N/A	1.0 (0.8)	0.9 (1.1)	0.8 (0.9)	0.7 (1.0)
MHS	1.1 (0.1)	2.6 (0.5)	2.4 (0.4)	2.6 (0.4)	2.2 (0.4)

## Data Availability

The data used and/or analyzed within the present study are available from the corresponding author on reasonable request.

## Supplementary Materials

The following supporting information can be downloaded at: https://www.mdpi.com/article/10.3390/diagnostics12061378/s1: Supplementary File S1: Table S1: Ultrasonographic landmarks and probe positions. Supplementary File S2: Tables S2 and S3: Corresponding *p* values for Table 2 and Table 3; Table S4: ROC analysis of ultrasonographic parameters.
