# Supplementary material for: Sonographic Evaluation of Muscle Echogenicity for the Detection of Intensive Care Unit-Acquired Weakness: A Pilot Single-Center Prospective Cohort Study
Source: Diagnostics (Basel). 2022 Jun 2;12(6):1378. doi: 10.3390/diagnostics12061378 (PMC9221760; doi:10.3390/diagnostics12061378)
Supplement: Supplementary file 1 [file diagnostics-12-01378-s001.zip › Supplementary File S1.pdf]

Table S1. Ultrasonographic landmarks and probe positions.

**Assessed muscles and positioning of the ultrasound probe using anatomic landmarks**	**Examples of corresponding ultrasound images**
**Upper extremity muscles**	
**Musculus biceps brachii**<br><br>Half of the way between Tuberculum majus humeri and Fossa olecrani	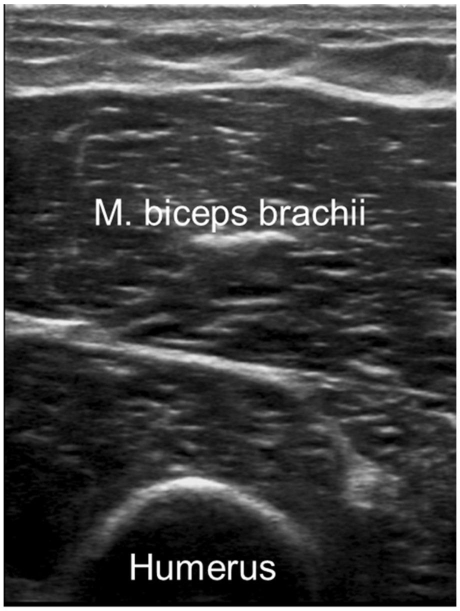
**Musculus brachioradialis**<br><br>Proximal third part of the way between the anatomical landmarks of the Lateral epicondyle humeri and the Styloid process of radius	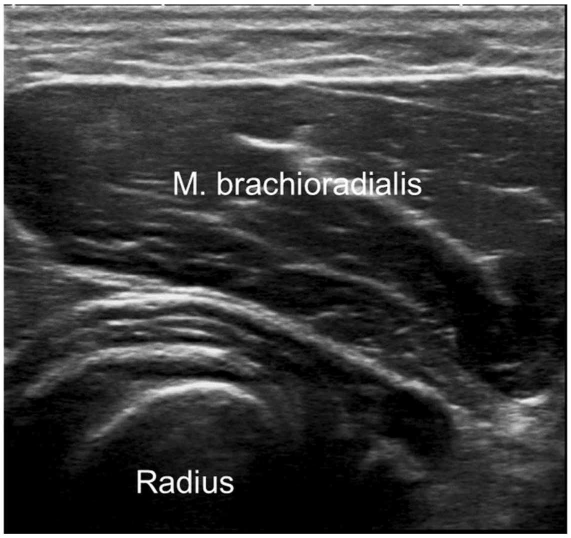

Assessed muscles and positioning of the ultrasound probe using anatomic landmarks	Examples of corresponding ultrasound images
**Lower extremity muscles**	
**Musculus rectus femoris of the quadriceps femoris**<br><br>Distal third of the way between anterior superior iliac spine and upper pole of the patella	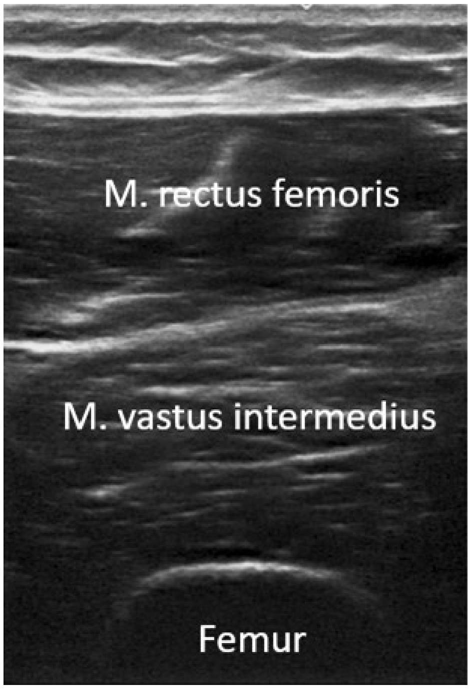
**Musculus tibialis anterior**<br><br>Five centimeters distal and one centimeter lateral from the distal pole of the patella	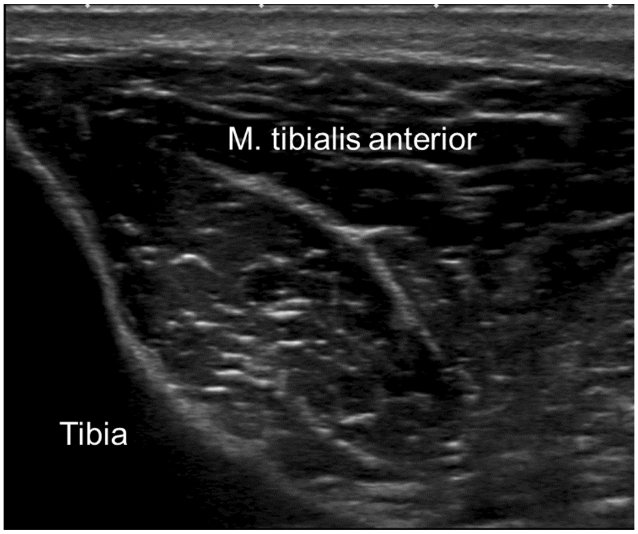
