# Supplementary material for: Sonographic Evaluation of Muscle Echogenicity for the Detection of Intensive Care Unit-Acquired Weakness: A Pilot Single-Center Prospective Cohort Study
Source: Diagnostics (Basel). 2022 Jun 2;12(6):1378. doi: 10.3390/diagnostics12061378 (PMC9221760; doi:10.3390/diagnostics12061378)
Supplement: Supplementary file 1 [file diagnostics-12-01378-s001.zip › Supplementary File S2.pdf]

Table S2. P values of mean value comparisons of the parameters of muscle echogenicity displayed in table 2. Statistically significant p values ($p \leq 0.006$) are printed in bold. GS: greyscale value. HS: Heckmatt scale. ICU-AW: intensive care unit - acquired weakness. N/A: value not available. GSSD: greyscale standard deviation. SFT: subcutaneous fat layer thickness.

**p values**	**Healthy controls vs ICU-AW+ day 3 / day 10**	**Healthy controls vs ICU-AW- day 3 / day 10**	**ICU-AW+ day 3 vs ICU-AW- day 3**	**ICU-AW+ day 10 vs ICU-AW- day 10**
**Biceps brachii left**				
GS	0.02 / 0.01	0.08 / 0.11	0.54	0.13
z-Score	N/A	N/A	0.54	0.14
HS	**<0.001 / <0.001**	**<0.001 / <0.001**	0.06	0.05
GSSD	0.70 / 0.39	0.96 / 0.28	0.43	0.76
SFT (cm)	0.06 / 0.05	0.03 / 0.13	0.92	0.15
**Biceps brachii right**				
GS	0.01 / 0.03	0.02 / 0.07	0.88	0.22
z-Score	N/A	N/A	0.88	0.66
HS	**<0.001 / <0.001**	**<0.001 / <0.001**	0.37	0.16
GSSD	0.09 / 0.24	0.13 / 0.73	0.87	0.48
SFT (cm)	0.02 / 0.08	0.03 / 0.11	0.58	0.38
**Brachioradialis left**				
GS	**0.003** / 0.02	0.01 / 0.09	0.80	0.46
z-Score	N/A	N/A	0.79	0.47
HS	**<0.001 / <0.001**	**<0.001 / <0.001**	0.26	0.04
GSSD	0.45 / 0.75	0.79 / 0.89	0.23	0.82
SFT (cm)	0.19 / 0.14	0.15 / 0.66	0.77	0.25
**Brachioradialis right**				
GS	0.01 / 0.02	0.02 / 0.06	0.53	0.69
z-Score	N/A	N/A	0.46	0.70
HS	**<0.001 / <0.001**	**<0.001 / <0.001**	0.14	0.02
GSSD	0.10 / 0.81	0.54 / 0.16	0.09	0.30
SFT (cm)	0.21 / 0.10	0.34 / 0.75	0.70	0.14
**Rectus femoris of the Quadriceps femoris left**				
GS	0.09 / 0.08	0.08 / 0.12	0.48	0.46
z-Score	N/A	N/A	0.48	0.48
HS	**<0.001 / <0.0001**	**<0.001 / 0.001**	0.07	0.13
GSSD	0.31 / 0.52	0.25 / 0.88	1.0	0.59
SFT (cm)	0.01 / 0.04	0.51 / 0.40	0.08	0.20
**Rectus femoris of the Quadriceps femoris right**				
GS	0.39 / 0.17	0.52 / 0.16	0.81	0.98
z-Score	N/A	N/A	0.81	0.99
HS	**<0.001 / <0.001**	**<0.001 / <0.001**	0.49	0.09
GSSD	0.01 / 0.20	**<0.001** / 0.08	0.40	0.65
SFT (cm)	0.01 / **0.003**	0.14 / 0.33	0.51	0.11
**Tibialis anterior left**				
GS	0.01 / 0.02	**0.002 / 0.006**	0.35	0.70
z-Score	N/A	N/A	0.35	0.70
HS	**<0.001 / <0.001**	**<0.001 / <0.001**	0.09	0.21
GSSD	0.48 / 0.67	0.98 / 0.11	0.47	0.15
SFT (cm)	0.29 / 0.70	0.90 / 0.88	0.25	0.61
**Tibialis anterior right**				
GS	**<0.001 / 0.002**	0.01 / **0.001**	0.33	0.60
z-Score	N/A	N/A	0.33	0.60
HS	**<0.001 / <0.001**	**<0.001 / <0.001**	0.80	**0.001**
GSSD	0.46 / 0.90	0.95 / 0.56	0.58	0.54
SFT (cm)	0.22 / 0.02	0.31 / 0.12	0.59	0.14

Table S3. Corresponding p values to parameters given in Table 3. Statistically significant p values ($p \leq 0.006$) are printed in bold. The results are rounded to the first decimal place. ICU-AW: intensive care unit - acquired weakness. MGS: global mean greyscale score. MHS: global mean Heckmatt score. MZS: global mean z-score. N/A: data not available.

p values	Healthy controls vs ICU-AW+ day 3 / day 10	Healthy controls vs ICU-AW- day 3 / day 10	ICU-AW+ day 3 vs ICU-AW- day 3	ICU-AW+ day 10 vs ICU-AW- day 10
MGS	**0.002** / 0.01	0.02 / 0.04	0.79	0.73
MZS	N/A	N/A	0.73	0.73
MHS	**<0.001 / <0.001**	**<0.001 / <0.001**	0.07	**0.006**

Table S4. ROC analysis. Statistically significant p values ($p < 0.05$) are printed in bold. AUC: area under the curve. MGS: global mean greyscale score. MHS: global mean Heckmatt score. MZS: global mean z-score.

Parameter	AUC	Standard error	p value	95% confidence interval
**MGS**				
Day 3	0.53	0.1	0.79	0.33 to 0.72
Day 10	0.53	0.1	0.76	0.34 to 0.72
**MZS**				
Day 3	0.54	0.1	0.72	0.34 to 0.73
Day 10	0.53	0.1	0.78	0.34 to 0.72
**MHS**				
Day 3	0.64	0.1	0.15	0.46 to 0.83
Day 10	0.79	0.08	**0.003**	0.64 to 0.94
